# Use of Aprotinin in a Patient With a Renal Transplant for Redo Coronary Artery Bypass Graft Surgery

**DOI:** 10.7759/cureus.108582

**Published:** 2026-05-10

**Authors:** Hafiz Muhmmad Shurjeel Latif

**Affiliations:** 1 Anaesthesiology, Tallaght University Hospital, Dublin, IRL

**Keywords:** aprotinin, coronary artery bypass grafting (cabg), glyceryl trinitrate, monoclonal gammopathy of undetermined significance (mgus), non-st segment elevation myocardial infarction (nstemi), patent foramen oval

## Abstract

We report a case of a high-risk redo coronary artery bypass graft (CABG) surgery performed on a 58-year-old patient admitted with a non-ST-segment elevation myocardial infarction (NSTEMI) and heart failure. The patient's medical history is significant for previous mechanical aortic valve replacement due to aortic stenosis, prior CABG, and closure of a patent foramen ovale (PFO). Additional comorbidities include type I diabetes mellitus, a history of right temporo-parietal ischaemic stroke, fatty liver disease, monoclonal gammopathy of unknown significance (MGUS), and renal transplantation. In this case, aprotinin infusion was utilised intraoperatively to manage perioperative bleeding. We focused on evaluating the effects of aprotinin on renal function and postoperative renal indices.

## Introduction

Renal transplantation is the definitive management strategy for end-stage renal disease, and a transplanted kidney can last for 10-25 years [1]. Patients with renal disease are at increased risk of developing coronary artery disease and thus may present for cardiac surgery. A major management goal in such cases is maintaining renal function, particularly in patients with a transplanted kidney. However, acute kidney injury following cardiac surgery is a well-recognised complication, and perioperative bleeding is a proven contributory factor [2]. Consequently, strategies to reduce perioperative bleeding are important in the overall management of renal transplant patients presenting for cardiac surgery.

Redo cardiac surgery not only increases morbidity and mortality but also contributes towards significant perioperative bleeding, with up to 50% of patients requiring blood transfusion [3].

Aprotinin is a serine protease inhibitor used in cardiac surgery to reduce blood loss and the need for transfusion [4]. It has had a complicated past; initial studies suggested that it contributed to an increased rate of post-operative renal complications; however, further analysis and study have since allayed this concern [5].

We report the case of a patient with a renal transplant who required redo sternotomy for coronary artery bypass graft surgery and who received aprotinin at half the usual dose as an effective strategy to reduce blood loss during surgery.

## Case presentation

A 58-year-old man presented following a non-ST-segment elevation myocardial infarction (NSTEMI) with acute decompensated heart failure. Initial medical management included anticoagulation (heparin infusion), dual antiplatelet therapy, and a glyceryl trinitrate (GTN) infusion. Despite this management, the patient continued to have intermittent chest pain, with worsening pulmonary oedema and an increasing demand for supplemental oxygen.

The patient had a medical history of previous mechanical aortic valve replacement (for aortic stenosis), coronary artery bypass grafting (CABG), and closure of a patent foramen ovale (PFO) five years previously. That surgery had been complicated by post-operative renal failure, which required renal replacement therapy and ultimately resulted in the need for a renal transplant, which was performed two years following the initial surgery.

Prior to this hospital admission, the patient's baseline serum creatinine level was 150 μmol/L, and urine output typically ranged from 1.5 to 2 L/day. Owing to the presence of the mechanical valve, the patient was on warfarin and was receiving immunosuppressive medications (tacrolimus, prednisolone, and mycophenolate) for his renal transplant. Additionally, the patient had several other comorbidities, including type I diabetes mellitus, a previous right temporo-parietal ischaemic stroke, fatty liver, and monoclonal gammopathy of undeterminate significance (MGUS).

Preoperatively, coronary angiography revealed severe triple vessel disease affecting the left coronary artery main stem (LMS), the left anterior descending artery (LAD), and the left circumflex artery (LCx). Trans-thoracic echocardiography showed a left ventricular ejection fraction (LVEF) of 50% with hypokinetic infero-septal and antero-septal walls, a dilated left atrium, reduced global systolic function of the right ventricle, and mild to moderate tricuspid regurgitation. The mechanical prosthetic aortic valve was functioning effectively with a small paravalvular leak.

During the admission, the patient developed hospital-acquired pneumonia and was initiated on piperacillin-tazobactam. Additionally, the patient contracted COVID-19 and required isolation and high-flow nasal oxygen therapy. The baseline creatinine level increased to 210 μmol/L, and the patient was commenced on intravenous furosemide. Given the presence of a mechanical aortic valve, heparin infusion was commenced.

In planning for surgery, a multidisciplinary team meeting was convened with the cardiothoracic surgeon, anaesthetist, and nephrologist. The primary focus was to address the pertinent issues, namely, the potential risk of bleeding in a patient who was anticoagulated but required a redo sternotomy, and to establish the most effective management strategy to safeguard the transplanted kidney from further harm. After thorough deliberation and mutual consensus, it was determined that the patient should receive aprotinin to mitigate the risk of excessive blood loss. However, due to nephrotoxic concerns, the decision was made to administer only half of the standard dose. It was also established that the transfusion trigger will be a haemoglobin of 9 g/dL.

CABG was performed using a midline redo sternotomy. The left internal mammary artery (LIMA) was grafted to the LAD; the OM was too small to be grafted. The patient was given 1 million units of aprotinin pre-cardiopulmonary bypass (CPB), and an additional 1 million units were added to the CPB circuit. A maintenance infusion of 0.5 million units per hour was initiated after bypass was established, resulting in a cumulative total of 3 million units administered. Our patient's baseline activated clotting time (ACT) was 101 seconds. To achieve the target ACT of >480 seconds, the patient received 30,500 IU of unfractionated heparin (390 IU/kg). Additionally, a stress dose of 100 mg of hydrocortisone was administered at the time of induction.

The total CPB time was 100 minutes, with a cross-clamp time of 89 minutes; 500 mg of protamine was given to reverse the effects of heparin. The ACT post-protamine was 128 seconds.

Pre-CPB transoesophageal echocardiography showed moderate LV systolic function, with no regional wall motion abnormality. The mechanical aortic valve was well seated, with no paravalvular leak and a peak gradient of 39 mmHg. Intraoperative estimated blood loss was approximately 570 mL. Due to the transfusion trigger of haemoglobin of 9 g/dL and the patient's baseline haemoglobin of 9.3 g/dL, the patient received 3 units of red blood cell concentrate (RCC) and 470 mL of salvaged cell-saver blood. Additional blood products, including 4 units of fresh frozen plasma, 3 g of fibrinogen, and 2 pools of platelets, were given to address coagulopathy and maintain haemostasis post CPB. The urine output was 700 mL during the course of the procedure. Intra-operative hyperkalaemia was managed using dextrose 50% with insulin infusion and filtration while on CPB. The patient was commenced on a dobutamine infusion of 1 mcg/kg/min and a milrinone infusion of 0.05 mcg/kg/min to facilitate coming off CPB. Post-CPB echocardiography was unremarkable.

The patient was transferred to the cardiac intensive care unit and extubated in accordance with local protocols. There was minimal output from the chest drains (10-20 mL/hr) and were removed as per local guidelines. Ongoing renal monitoring continued 8 hourly for the first 24 hours and daily thereafter. The maximum urea level was 19.1 mmol/L and creatinine 195 μmol/L on day 1 post-op, with urine output of 0.5-1 mL/kg/hr. On day 2 post-op, the maximum urea level was 22 mmol/L, and the creatinine level was 230 μmol/L. Furosemide 40 mg IV twice daily was commenced to maintain UOP at 0.5-1 mL/kg/hr. Serum creatinine peaked on D4 at 313 μmol/L and urea on D6 at 33.7 mmol/L. IV furosemide was increased to 40 mg three times daily to maintain UOP. A renal consult was sought, and the current management continued. The subsequent renal parameters indicated a declining trend (Table 1).

**Table 1:** Renal Indices Post Redo Cardiopulmonary Bypass

Post-op Day	Urea (Reference Range: 2.5–7.8 mmol/L)	Creatinine (Reference Range: 59–104 μmol/L)
1	19.1	195
2	21.9	229
3	27.0	273
4	29.6	313
5	32.8	302
6	33.7	261
7	32.7	253
8	Not done	Not done
9	30.9	206
10	26.2	198
11	20.6	176
12	16.2	165

The patient developed a leg wound infection during the course of the stay and was commenced on IV and then oral antibiotics. The blood glucose was also difficult to manage, requiring multidisciplinary input. After settling the acute phase, the patient was transitioned to ward-level care on day five post-op and was successfully discharged home on day 17 post-operatively.

## Discussion

Aprotinin is a non-selective serine protease inhibitor, initially derived from bovine sources in the 1930s. It exerts non-specific inhibition of trypsin-like serine proteases, including trypsin, plasmin, plasma and tissue kallikreins and coagulation factor XII [4]. After the Lancet publication in 1987 and subsequent popularity, evidence of adverse effects, particularly renal impairment, emerged, prompting the voluntary withdrawal of the product from the market and the revocation of its licence by the Marketing Authorisation Holder (MAH) and the European Medicines Agency (EMA) in 2007-08 [6,7].

Aprotinin was subsequently reintroduced into medical practice in 2016 following the discovery of discrepancies in the initial data. The Nordic Aprotinin Patient Registry (NAPaR) was established to collect data from 83 cardiac centres where aprotinin was used. These data were then analysed as part of a non-interventional, post-authorisation safety study (PASS). The study's findings indicated that there was no discernible evidence of increased mortality or renal injury among patients who had been exposed to aprotinin therapy for various cardiac procedures, including isolated CABG and other cardiac surgeries, even those of a complex nature carrying a high risk of death or significant blood loss [8].

Worner et al. conducted a study on mice to investigate the renal adverse effects of aprotinin. Their findings concluded that when aprotinin was administered in reduced dosages, there was no apparent evidence of acute kidney injury [4]. These results could reasonably be applied to humans, given the similar renal handling of aprotinin. To compensate for rapid renal clearance, it is administered as a continuous infusion [9].

Our patient was at increased risk of bleeding, with risk factors including redo surgery and renal impairment. Both redo sternotomy and renal impairment are associated with an increased risk of bleeding, with renal impairment alone contributing up to a 35% increased risk of haemorrhage [10]. However, our patient’s blood loss during the surgery was 570 mL and was comparable to the blood loss during coronary artery bypass graft surgery, as identified by Bjessmo et al. (median pre-operative bleeding: 550 mL, range: 200-1700 mL) [11].

## Conclusions

This case may contribute to the growing body of evidence that aprotinin can be safely used in patients presenting for redo sternotomy to reduce bleeding. Further study should focus on the optimal dosing strategy. This case further highlights the importance of a multidisciplinary approach to complex patients.
